# Empirically derived dietary patterns and constipation among a middle-aged population from China, 2016–2018

**DOI:** 10.1186/s12937-019-0512-9

**Published:** 2019-12-26

**Authors:** Li Li, Ai-Ping Huang, Li-Qin Wang, Xiao-Long Yu

**Affiliations:** 1 Graduate Office, The First Hospital of Lanzhou University, Chengguan district, Lanzhou, 730000 Gansu China; 2 Department of Blood donation service, Blood Center of Zhejiang Province, Xiacheng district, Hangzhou, 310006 Zhejiang China; 3 Department of Rheumatology, Affiliated Hospital of Gansu University of Traditional Chinese Medicine, Chengguan district, Lanzhou, 730000 Gansu China; 4 Department of Nutrition, Zhejiang Hospital, Xihu district, Hangzhou, 310013 Zhejiang China

**Keywords:** Dietary patterns, Constipation, Chinese, Cross-sectional study

## Abstract

**Background:**

The association of dietary patterns with constipation is not well established, particularly in Chinese population. Therefore, the present study aimed to determine the relationship between dietary patterns and the risk of constipation in a middle-aged Chinese population.

**Methods:**

A total of 2267 participants aged 45–59 years were recruited in Hangzhou city, the capital of Zhejiang Province, east China from August 2016 to October 2018. Dietary intake was estimated using a semi-quantitative food frequency questionnaire (FFQ) containing 138 food items. Constipation was defined using the Rome II criteria. Multivariate logistic regression was used to examine the association between dietary patterns and the risk of constipation.

**Results:**

Three major dietary patterns were extracted by factor analysis and labeled as the traditional southern Chinese, Western and grains-vegetables patterns. The prevalence of constipation in our study population was 13.28%. Compared with the participants in the lowest quartile, the participants in the highest quartile of the traditional southern Chinese pattern were associated with reduced odds of constipation (odd ratios (OR) = 0.79; 95%confidence interval (CI): 0.626–0.981; *P* < 0.05), after adjusting for confounding variables. In contrast, we found no significant associations between the Western and grains-vegetables patterns and the risk of constipation (*P* > 0.05).

**Conclusions:**

Our study demonstrated that the traditional southern Chinese pattern was associated with a reduced risk of constipation. Further longitudinal studies are needed to confirm our findings.

## Background

Constipation is a common functional gastrointestinal disorder in clinical practice, with a prevalence ranging from 5 to 20% of the population worldwide [1]. In Japan, the prevalence of constipation, which was defined as≤3 bowel movements weekly, seems to be relatively high (approximately 6%~ 25% of the general population) [2]. In contrast, constipation in the Western countries has also attracted increasing attention given its high prevalence in children, estimated at 10%~ 25% [3]. It is well established that constipation is a multifactorial chronic disease that may be related to some factors, including age, gender, genetic, psychosocial, socio-economic status, smoking status, alcohol intake, physical activity, and dietary factors [4, 5].

At present, diet has been recognized as a modifiable factor that plays a key role in the development of constipation [6]. A considerable number of epidemiological studies have reported the associations between diet and constipation, concentrating on the effects of individual nutrients or foods and food groups [7–9]. However, in real life, people do not consume certain foods or single nutrients, but rather mixed food that contains various nutrients [10]. Against this background, dietary pattern analysis, taking into account the interactions and inter-correlations between nutrients and foods as well as their cumulative effects on health, has gained attention in the study of the association between diet and disease [11]. Moreover, it also potentially facilitates nutritional recommendations [12].

Over the last decade, many epidemiological studies have been performed to elucidate the association of dietary patterns with chronic diseases, such as obesity, hypertension and type 2 diabetes mellitus [12–15]. However, data on the association of dietary patterns with the risk of constipation are sparse. To date, only one cross-sectional study has investigated dietary patterns in relation to the risk of functional constipation in Japanese women aged 18–20 years [16]. Furthermore, to our knowledge, no previous study has investigated the relationship between dietary patterns and the prevalence of constipation in Chinese population. In view of this, the purpose of the present study was to derive the major dietary patterns existing in a middle-aged Chinese population and to assess their associations with the risk of constipation.

## Subjects and methods

### Study population

This study was conducted in Hangzhou city, capital of Zhejiang Province, as a part of Hangzhou Nutrition and Health survey, from August 2016 to October 2018. This study sample was taken from ten areas (Shangcheng, Xiacheng, Bingjiang, Jianggan, Xihu, Gongshu,Yuhang, Xiaoshan, Fuyang and Linan) and three counties (Tonglu, Chunan, Jiande) by a stratified cluster random-sampling method. We chose two residential village or community from every county or area randomly, according to resident health records, with participants aged 45–59 years residing in the selected villages or communities. In brief, detailed information about the study design and data collection has been described in our previous study [10]. A total of 2437 eligible participants (1440 male, 1294 female; aged 45–59 years) were invited to attend a health examination at the Medical Center for Physical Examination, Zhejiang Hospital, where the participant was face-to-face interviewed by a trained interviewer using written questionnaires. For this analysis, we excluded 32 participants who provided incomplete anthropometric information, 66 participants who provided missing information on diet or had more than 15 items blank on the FFQ, 41 participants who reported implausible energy intakes(< 600 or > 4000 kcal/d), 17 who did not provide a stool sample, 12 participants with self-reported the family history of constipation, and 2 participants with self-reported the family history of cancer. After all these exclusions, a total of 2267 participants were included in the final analysis. All participants provided their written informed consent before data collection.

### Assessment of dietary intake

Usual dietary intake was assessed using a validated semi-quantitative FFQ that listed 138 food items regularly ingested by Chinese [10]. Participants were requested to complete the questionnaire regarding their food intake frequency, using local weight units (1 liang = 50 g) or household measures (cups) over the preceding year. Each food item had nine options for frequency that ranged from “never” to “3 times/d” and three options for portion size. Details of the dietary survey have been previously published [12]. Moreover, dietary fiber intake was estimated by the modified Prosky method (Science and Technology Agency, 2000) from the intake of 74 fiber-containing foods in this FFQ. In the present study, dietary fiber intake data for participants were calculated by the Chinese food ingredient list(2018). Finally, the frequency category for each food item was converted into g/d in the following analysis.

### Identification of dietary patterns

First, we collapsed 138 food items from the FFQ into 30 predefined food groups (in Table 1) based on their similarity of type of food and nutrient composition in a middle-aged Chinese population [10]. Then, we conducted the factor analysis (principal component) on the 30 predefined food groups to derive the major dietary patterns. Second, to achieve a simpler structure with greater interpretability, the factors were rotated by an orthogonal transformation (varimax rotation) in SPSS version 23.0. To determine the number of factors, the eigenvalue, scree plot and factor interpretability were applied in our analyses [17]. After evaluating the eigenvalues, the scree plot test, and interpretability, factors with an eigenvalues ≥1.5 were retained. Finally, food groups with factor-loading absolute values> 0.4 were considered to significantly contribute to each pattern in present study. Factor scores were categorized into quartiles (Q1 represented a low intake of the food pattern; Q4 represented a high intake of the food pattern).

**Table 1:** Food grouping used in the dietary pattern analyses

Food groups	Food items
Refined grains	Rice, porridge, rice in soup, noodles,instant noodles,steamed bun, wonton, dumplings, white breads, toasted bread
Whole grains	Corn, sorghum, millet, oats
Tubers	Sweet potato, potato, taro
Vegetables	Wild vegetables, green vegetable, spinach, green peppers, tomato Chinese cabbage, radish, cucumer, eggplant
Fruit	Apple,pears, peach, apricots,cherries,grapes,bananas,cantaloupe,watermelon, oranges, grapefruit, kiwi,strawberries and et al
Pickled vegetables	Salted vegetables, Chinese sauerkraut
Mushrooms	Mushroom, shiitakes, enoki
Red meat	Pork, mutton, beef
Poultry and organs	Chicken, duck, liver, animal blood
Processed and cooked meat	Ham and sausage, sauced pork, roast duck
Fish and shrimp	Fish, shrimp
Eggs	Duck eggs, chicken eggs
Seafood	Sea fish, shrimp, crab,squid, jellyfis, shellfish
Bacon and salted fish	Salted meat and duck, salted fish
Salted and preserved eggs	Salted duck and chicken eggs, preserved eggs
milk	Liquid milk, milk powder, yoghurt
Cheese	Cheese
Soya bean and its products	Tofu, dried bean curd, soy milk
Miscellaneous bean	Mung beans, red beans, hemp beans
Fats	Lard, butter
Vegetable oil	Soybean oil, tea oil, rapeseed oil, olive oil
Fast foods	KFC, Mcdonald,fried dough sticks and twists, fried cakes,pizza
Nuts	Walnut, peanuts, almonds, melon seeds
Snacks	Cookies, sachima, bread, cake, ice cream, candy, sweets,potato chips, shrimp roll, popcorn
Chocolates	Chocolates
Honey	Honey, hydromel
Drinks	Coca-cola, sprite, fruit and vegetable drink, fruits juice
Alcoholic beverages	Beer, fruit wine, grape wine
Tea	Tea,scented tea, wong Lo Kat
Coffee	Coffee

### Assessment of blood pressure

Participant’s blood pressure was measured by a trained nurse after a 5–10 min rest in the sitting position with a standard mercury sphygmomanometer. The blood pressure was measured three times, and the average of three measurements was considered as the participant’s blood pressure.

### Assessment of anthropometric measurements

Height (cm) and weight (kg) were measured to the nearest 0.1 cm and 0.1 kg respectively, using a digital electronic scale (Tanita) with the participants wearing light clothing and no shoes. Body mass index (BMI) was calculated as weight (kg) divided by the square of height(m^2^). Waist circumference (WC) was measured to the nearest 0.1 cm at the natural waist (minimal circumference between umbilicus and xiphoid process) [18]. All anthropometric measurements were performed by well-trained nurses to use standardized procedures.

### Assessment of other variables

Data on physical activity was collected by means of the International Physical Activity Questionnaire (IPAQ), and expressed as metabolic equivalent hours per week (MET-h/week) [19]. Additional information such as age, gender, smoking status, educational level, income and usage of medication was obtained with a validated self-reported questionnaire. Smoking status was categorized as never smoker, current smoker, and former smoker. Educational level was divided into three categories: primary school or below, middle and high school, junior college or above. The usage of medication, such as desert cistanche, duphalac, macrogol 4000 powder for oral solution was collected by a questionnaire survey. In addition, total energy intake was estimated using a validated semi-quantitative FFQ, expressed in kilocalorie per day (kcal/day).

### Definition of constipation

According to the ROME II criteria introduced in 2000 [20], participants those who met two or more following diagnostic criteria were defined as having constipation:(1)straining; (2) lumpy or hard stools; (3) incomplete evacuation;(4) sensation of obstruction or blockage;(5) fewer than three defaecations per week. The most common constipation symptoms include abdominal pain or discomfort, hard stools, feeling of incomplete evacuation, excessive straining, sense of anorectal blockage, and the need for manual maneuvers [21]. Participants were asked to answer complete the following four questions in written questionnaire during the previous 12 months. The four questions are as follows:(1) do you strain during bowel movements? (2) do you feel a sensation of incomplete emptying after bowel movements? (3) how hard are your stools? (4) how many times do you usually have bowel movements every week?

### Definition of terms

Obesity was defined by BMI ≥ 28 kg/m2 and abdominal adiposity was defined as (male: WC ≥ 85 cm; female: WC ≥ 80 cm) [22]. Hypertension was defined as a systolic pressure of 140 mmHg or higher and/or a diastolic pressure of 90 mmHg or higher [23].

### Statistical analyses

The study participants were categorized according to quartile categories of the dietary pattern scores. Data were calculated across quartiles of each dietary pattern scores, and presented as means±standard deviation (SD) for continuous variables and number (proportion) for categorical variables. If data are normal distributed variables, we used independent-samples T test to assess the significant differences in continuous variables. If not, the Mann-Whitney test was required. Besides, the chi-squared test was used to examine the difference between categorical variables. Binary logistic regression models were applied to evaluate the associations of major dietary patterns with the risk of constipation, adjusting for potential confounders. The first model was adjusted for gender (male/female) and age (years). The second model was further adjusted for education level(<high school, high school, >high school), physical activity level (light, moderate, heavy), smoking status (never, current, former), alcohol intake, income, BMI (continuous) and total energy intake; The third model was additionally adjusted for total fiber intake. All statistical analyses were performed using version 23.0 of the SPSS software package (SPSS Inc., Chicago, IL, USA), and a 2-sided *P-value* < 0.05 was considered statistically significant.

## Results

Overall prevalence of constipation in this population was 13.28% (301/2267). General characteristics of participants with and without constipation are shown in Table 2 (*n* = 2267). There were statistical differences for age, education, income, total energy intake and the prevalence of hypertension between participants with and without constipation (*P* < 0.05). Participants with constipation were significantly younger (50.37 ± 5.16 vs 52.65 ± 5.60), lower educational level (19.9% vs 25.7%) and economic income (34.5% vs 29.6%) and and higher total energy intake(1908.2 ± 186.3 vs 1783.1 ± 155.4) and prevalence of hypertension(21.9% vs 16.4%) than those without constipation.

**Table 2:** General characteristics of participants with and without constipation

Variables	Participants with constipation (*n* = 301)	Participants without constipation (*n* = 1966)	Significance^*^
Demographic			
Age (years)	50.37 ± 5.16	52.65 ± 5.60	*P <* 0.001
Gender			
Male	139(46.2)	993(50.5)	χ^2^ = 1.957 *P* = 0.162
Female	162(53.8)	973(49.5)	
Smoking status (%)			
Never	173(57.5)	1217(61.9)	χ^2^ = 2.172 *P* = 0.338
Former	31(10.3)	185(9.4)	
Current	97(32.2)	564(28.7)	
Education (%)			
< High school	24(8.0)	187(9.5)	χ^2^ = 6.240 *P* < 0.05
High school	217(72.1)	1274(64.8)	
> High school	60(19.9)	505(25.7)	
Monthly income per person(%)			
≤ 2500 (RMB)	202(34.5)	582(29.6)	χ^2^ = 60.351 *P* < 0.001
2500–4000 (RMB)	218(50.2)	987(50.2)	
> 4000 (RMB)	33(15.3)	397(20.2)	
Physical activity(%)			
Light	194(64.5)	1339(68.1)	χ^2^ = 3.767 *P* = 0.152
Moderate	76(25.2)	401(20.4)	
Heavy	31(10.3)	226(11.5)	
Total energy intake (Kcal/d)	1908.2 ± 186.3	1783.1 ± 155.4	*P* < 0.05
Obese (%)			
Yes	49(16.3)	265(13.5)	χ^2^ = 1.715 *P* = 0.190
No	252(83.7)	1701(86.5)	
Hypertension(%)			
Yes	66(21.9)	322(16.4)	χ^2^ = 5.665 *P* = 0.017
No	235(78.1)	1644(83.6)	

Categorical variables are presented as sum and percentages, and continuous variables are presented as Mean ± SD. Abbreviation: RMB: Ren Min Bi.^*^P values for continuous variables (independent-samples T or Mann-Whitney test) and for Categorical variables (chi-square test)

Both the Kaiser-Meyer-Olkin index (0.703) and Bartlett’s test (*P* < 0.001) showed that the correlation among the variables was sufficiently strong for a factor analysis [10]. Three major dietary patterns were extracted by factor analysis. The first pattern, namely traditional southern Chinese dietary pattern, was characterized by high intake of refined grains, vegetables, fruits, pickled vegetables, fish and shrimp, eggs, bacon and salted fish, salted and preserved eggs, milk, soya bean and its products, miscellaneous bean, fats, drinks. The second pattern, namely Western dietary pattern, was characterized by high intake of red meats, poultry and organs, processed and cooked meat, seafood, cheese, fast foods, snacks, chocolates, alcoholic beverages, coffee. The third pattern, namely grains-vegetables dietary pattern, was characterized by high intake of whole grains, tubers, vegetables, mushrooms, vegetable oil, nuts, honey, tea. These factors explained 10.3, 8.5 and 6.8% of the dietary intake variance, respectively. Besides, the factor-loading matrixes for these dietary patterns are shown in Table 3.

**Table 3:** Factor-loading matrix for the three dietary patterns^*^

Food groups	Dietary patterns		
	Traditional southern Chinese	Western	Grains-vegetables
Refined grains	0.411	–	–
Whole grains	–	–	0.534
Tubers	–	–	0.471
Vegetables	0.407	–	0.638
Fruit	0.462	–	–
Pickled vegetables	0.509	–	–
Mushrooms	–	–	0.664
Red meat	–	0.563	–
Poultry and organs	–	0.502	–
Processed and cooked meat	–	0.520	–
Fish and shrimp	0.486	–	–
Eggs	–	0.346	–
Seafood	–	0.417	–
Bacon and salted fish	0.529	–	–
Salted and preserved eggs	0.414	–	–
Milk	0.360	–	
Cheese	–	0.315	
Soya bean and its products	0.400	–	–
Miscellaneous bean	0.414	–	–
Fats	0.447	–	
Vegetable oil	–	–	0.392
Fast foods	–	0.407	–
Nuts	–	–	0.303
Snacks	–	0.517	–
Chocolates	–	0.435	–
Honey	–	–	0.595
Drinks	0.472	–	
Alcoholic beverages	–	0.301	–
Tea	–	–	0.357
Coffee	–	0.390	–
Variance of intake explained (%)	10.3	8.5	6.8

^*^Absolute values< 0.4 were excluded for simplicity

General characteristics of study participants across quartiles of major dietary pattern scores are provided in Table 4. Compared with participants in the lowest quartile, those in the highest quartile of the traditional southern Chinese dietary pattern were more likely to be older, female, smokers, to use desert cistanche, and had lower prevalence of obesity and constipation, lower BMI and income, and higher education level. Besides, in comparison with the participants from the lowest quartile of the Western dietary pattern, those in the highest quartile were more likely to be younger, male, smokers, to use duphalac and other, and had higher prevalence of obesity and hypertension, and higher BMI, WC, WHR, educational level, income and total energy intake. Similarly, participants in the highest quartile of the grains-vegetables pattern were more likely to be older, female, never- smokers, and have lower BMI, WC, WHR, total energy intake and had significantly lower prevalence of obesity and hypertension and higher physical activity level.

**Table 4:** General characteristics of study participants across quartiles of the major dietary pattern scores

	Traditional southern Chinese dietary pattern score		P*	Western dietary pattern score		P*	Grains-vegetables dietary pattern score		P*
	Q1 (lowest) (*n* = 566)	Q4 (highest) (*n* = 567)		Q1 (lowest) (n = 567)	Q4 (highest) (n = 567)		Q1 (lowest) (*n* = 566)	Q4 (highest) (n = 567)	
Age (y)	50.1 ± 0.2	52.0 ± 0.3	**< 0.001**	51.5 ± 0.2	49.6 ± 0.2	**< 0.001**	50.2 ± 0.3	51.9 ± 0.3	**< 0.001**
BMI (kg/m^2^)	24.47 ± 2.88	23.88 ± 2.76	**< 0.05**	24.12 ± 2.93	24.96 ± 3.02	**< 0.05**	25.37 ± 2.83	24.31 ± 2.78	**< 0.01**
WC (cm)	87.49 ± 8.61	86.52 ± 8.53	0.152	84.53 ± 8.61	87.79 ± 8.50	**< 0.01**	87.96 ± 8.92	83.05 ± 8.13	**< 0.01**
WHR	0.88 ± 0.06	0.87 ± 0.05	0.236	0.87 ± 0.08	0.89 ± 0.06	**< 0.05**	0.89 ± 0.06	0.86 ± 0.08	**< 0.05**
Obesity (%)	92(16.3)	69(12.2)	**< 0.05**	62(10.9)	105(18.5)	**< 0.001**	89(15.7)	52(9.2)	**< 0.01**
Hypertension (%)	133(23.5)	144(25.4)	0.457	106(18.7)	134(23.6)	**< 0.05**	112(19.8)	81(14.3)	**< 0.05**
Constipation(%)	106(18.7)	71(12.5)	**< 0.01**	92(16.2)	103(18.2)	0.387	109(19.3)	103(18.2)	0.637
Gender (%)			**< 0.001**			**< 0.01**			**< 0.001**
Male	324(57.2)	265(46.7)		286(50.4)	342(60.3)		338(59.7)	217(38.3)	
Female	242(42.8)	302(53.3)		281(49.6)	225(39.7)		228(40.3)	350(61.7)	
Smoking status (%)			**< 0.001**			**< 0.001**			**< 0.01**
Never	454(80.2)	411(72.5)		400(70.5)	365(64.4)		410(72.4)	457(80.6)	
Current	58(10.2)	104(18.3)		64(11.3)	144(25.4)		64(11.3)	58(10.2)	
Former	54(9.6)	52(9.2)		103(18.2)	58(10.2)		92(16.3)	52(9.2)	
Education (%)			**< 0.001**			**< 0.01**			0.178
< High school	183(32.3)	105(18.5)		168(29.6)	113(19.9)		135(23.8)	136(24.0)	
High school	212(37.5)	171(30.2)		215(37.9)	245(43.2)		199(35.2)	172(30.3)	
> High school	171(30.2)	291(51.3)		184(32.5)	209(36.9)		232(41.0)	259(45.7)	
The average monthly income per person(%)			**< 0.01**			**< 0.001**			0.596
< 2000(RMB)	134(23.7)	187(33.0)		166(29.3)	115(20.3)		140(24.7)	138(24.4)	
2000–4000(RMB)	239(42.2)	229(40.4)		227(40.1)	218(38.4)		240(42.4)	227(40.0)	
> 4000(RMB)	193(34.1)	151(26.6)		173(30.6)	234(41.3)		186(32.9)	202(35.6)	
Physical activity (%)			**< 0.01**			**< 0.001**			**< 0.01**
Light	428(75.6)	382(67.4)		402(70.9)	467(82.4)		461(81.4)	431(76.0)	
Moderate	88(15.6)	115(20.3)		109(19.2)	71(12.5)		92(16.3)	100(17.6)	
Vigorous	50(8.8)	70(12.3)		56(9.9)	29(5.1)		13(2.3)	36(6.4)	
Drug usage									
Desert cistanche	36(6.4)	83(14.6)	**< 0.001**	48(8.5)	52(9.2)	0.675	66(11.7)	58(10.2)	0.440
Duphalac and other	82(14.5)	92(16.2)	0.417	64(11.3)	104(18.3)	**< 0.01**	74(13.1)	90(15.9)	0.181
Total energy intake (kcal/d)	2553.6 ± 184.6	2263.1 ± 198.2	**< 0.05**	2128.2 ± 265.7	2556.7 ± 198.6	**< 0.01**	2495.3 ± 203.5	2294.4 ± 195.3	**< 0.05**

Categorical variables are presented as sum and percentages, and continuous variables are presented as Mean ± SD. Abbreviation: BMI, Body mass index; WC, Waist circumference; WHR, Waist hip rate. * *P* values for continuous variables (Analysis of variance) and for Categorical variables (chi-square test); *P*-value < 0.05 was considered statistically significant

The association between major dietary patterns and the risk of constipation by logistic regression analysis is presented in Table 5. After adjustment for potential confounding variables, participants in the highest quartile of the traditional southern Chinese pattern scores had a lower risk of constipation (OR = 0.79; 95% CI: 0.626–0.981; *P* < 0.05) than those in the lowest quartile. In contrast, we found no significant associations between the grains-vegetables and Western patterns and risk of constipation (*P* > 0.05).

**Table 5:** Multivariable adjusted ORs and 95% CIs for constipation across the quartile (Q) categories of dietary pattern scores in Zhejiang Province, China

	Traditional southern Chinese pattern Score			Western pattern Score			Grains-vegetables Pattern Score		
	Q1	Q4	*p*	Q1	Q4	*p*	Q1	Q4	*p*
Model 1	1.00	0.52 (0.404,0.661)	0.000	1.00	1.41 (1.104,1.786)	0.02	1.00	0.65 (0.528, 0.814)	0.008
Model 2	1.00	0.68 (0.527,0.839)	0.000	1.00	1.11 (0.896,1.377)	0.29	1.00	0.85 (0.669, 1.072)	0.128
Model 3	1.00	0.79 (0.626,0.981)	0.04	1.00	1.09 (0.873,1.336)	0.59	1.00	0.93 (0.750, 1.195)	0.602

*OR* Odds ratio; *95%CI* 95% confidence interval. Model 1:adjusted for gender and age; Model 2: further adjusted for education level(<high school, high school, >high school),physical activity level (light, moderate, and heavy), smoking status (never, current, former), alcohol intake, BMI (continuous),and total energy intake; Model 3: additionally adjusted for total fiber intake. Q4: the highest quartile of dietary patterns, Q1: the lowest quartile of dietary patterns (reference); CI: confidence interval

## Discussion

Less is known about the influence of dietary patterns on the risk of constipation among Chinese populations. In the present study, we found that the traditional southern Chinese pattern was associated with a decreased risk of constipation in the middle-aged Chinese population. In contrast, no statistically significant associations were found between the grains-vegetables or Western patterns and the risk of constipation. To the best of our knowledge, this study is the first in the Chinese population to evaluate the association of dietary patterns with the risk of constipation.

In our analyses, the traditional southern Chinese dietary pattern was significantly associated with a lower prevalence of constipation. Our results are concordant with a previous study [16], which demonstrated that the Japanese traditional dietary pattern was significantly associated with a reduced risk of constipation. Several potential mechanisms have been proposed to explain this result. First, rice is a staple food in southern China. Previous studies have reported the favorable effect of rice on constipation in Asian population [7, 17, 24]. Second, vegetables and fruits have been related to providing large amounts of dietary fibers. Accumulating evidence has demonstrated that dietary fiber was inversely associated with constipation [9, 25]. Higher-fiber diet can increase stool weight, resulting in a decreased colon transit time, while poor-fiber diet induces constipation [26]. Third, Chillies are the main vegetable in southern China. Previous studies demonstrated that capsaicin-containing Chili, an irritant alkaloid, could induce abdominal burning and heighten rectal perception, influencing bowel transit [27]. Finally,the possible explanation of preventive effect of this pattern on constipation may contribute the higher intake of eggs. A previous study has shown an inverse association between egg consumption and the risk of constipation [28]. It is known that egg are the major source of digestible proteins. Yang et al.reported that digestible proteins could promote the formation of soft stools in elderly people [29].

In this study, the Western dietary pattern was characterized by high consumption of red meats, poultry and organs, processed and cooked meat, seafood, cheese, fast foods, snacks, chocolates, alcoholic beverages, and coffee. We did not observe the significant association between Western dietary pattern and the risk of constipation. The complex nature of this pattern may explain the null result to some extent. First, high consumption of meats, especially red meat, was associated with a decreased risk of constipation [30]. Second, low-to moderate consumption of coffee has been reported to be associated with a reduced risk of constipation [9]. An earlier study have shown that coffee is known to induce an increase in colonic motility limited to the rectosigmoid region with 4 min of ingestion and lasting at least 3 min [31]. In contrast, high intake of snacks and chocolates has also been reported to be associated with the increased risk of constipation [7]. Moreover, seafood contains large amounts of magnesium. Previous studies have demonstrated that the intake of magnesium is significantly associated with a decreased risk of constipation [16]. Finally, no significant association could be attributed to the lactose or sorbitol malabsorption in those particpants with high intake of snacks and fast foods, as well as higher CCK secretion (secondary to fat) that induces gastrocolic reflex [9]. Taken together, these possibilities could not be excluded in the present study.

Besides, we also did not find the significant association between the grains-vegetables pattern and the risk of constipation. This is inconsistent with previous studies in Japanese women, which showed an inverse association between Japanese traditional dietary pattern and the risk of constipation [7]. In our analyses, the grains-vegetables pattern was characterized by high intake of whole grains, tubers, vegetables, mushrooms, vegetable oil, nuts, honey, and tea. There are several possible explanations for the null result. First, whole grains, tubers and vegetables contain large amounts of dietary fiber. As stated above, dietary fiber has been regarded as an important determinant of constipation [9, 25]. Second, vegetables and mushrooms are rich in magnesium. Previous studies have demonstrated that the intake of magnesium is significantly associated with a decreased risk of constipation [16]. In contrast, high intake of tea has been found to be positively associated with the prevalence of constipation [32]. Moreover, an earlier research by Towers et al. reported that constipation is closely correlated with intake of energy [33]. however, it is worth noting that the participants in our study were predominately a group of middle and elderly Chinese, who consumed less foods, especially the high-energy foods. Elderly people are generally eating less than younger adults, so their energy intake is also lower than young people. Therefore, constipation in the elderly may be caused by low energy intake, which may reflect a low dietary intake. A previous study suggest that low dietary intake leads to reduced fecal volume and weight, which may lead to constipation [28]. Furthermore, the null association could also be due to the reverse causality. Participants diagnosed with functional constipation may be advised to change their dietary habits and lifestyles, such as increasing the intake of dietary fiber. These possibilities couldn’t be excluded in this study.

### Strengths and limitations

There are several strengths and limitations to our study. First, to authors’ knowledge, this is the first study to explore the relationship between dietary patterns and constipation in a middle-aged Chinese population. Second, diets were measured with the use of a semi-quantitative FFQ that were validate against multiple-day diet records; This tool enabled us to capture more reliable information on usual dietary intake of individuals during the preceding year. Meanwhile, the validity and reliability of this FFQ has also been verified elsewhere [12]. Third, in the current study, we have controlled for several known and proposed potential confounders in the statistical model. Nevertheless, several limitations of this study need to be acknowledged. First, the results do not show the causal association between dietary patterns and functional constipation, since this study was of cross-sectional design. Second, statistical methods (principal component analyses) used to derive dietary patterns, including grouping foods, choosing the number of factors and the labelling of dietary patterns, involve several subjective decisions [11]. Third, although the FFQ is a validated and well-established dietary assessment method, recall bias and underestimation or overestimation of food intake cannot be ruled out. Finally, the study participants were from the middle-aged population, not fully representative of the Chinese population, which may limit the generalisability of the findings.

## Conclusions

In conclusion, we found that the traditional southern Chinese pattern was significantly associated with a reduced risk of constipation in the middle-aged Chinese population. Our results further emphasize the importance of whole-diet in the prevention of constipation in a middle-aged Chinese population. However, given the cross-sectional nature of our study, these findings need be confirmed in further prospective studies.

## Data Availability

Not applicable.

## Abbreviations

ANOVA: Analysis of variance
BMI: Body mass index
CI: Confidence interval
DBP: Diastolic blood pressure
FFQ: Food frequency questionnaire
OR: Odds ratio
SBP: Systolic blood pressure
WC: Waist circumference
WHR: Waist-hip ratio
